# Cinnamaldehyde Mitigates Atherosclerosis Induced by High-Fat Diet *via* Modulation of Hyperlipidemia, Oxidative Stress, and Inflammation

**DOI:** 10.1155/2022/4464180

**Published:** 2022-06-21

**Authors:** Basma S. Ismail, Basant Mahmoud, Eman S. Abdel-Reheim, Hanan A. Soliman, Tarek M. Ali, Basem H. Elesawy, Mohamed Y. Zaky

**Affiliations:** ^1^Biochemistry Department, Faculty of Science, Beni-Suef University, P.O. Box 62521, Beni Suef, Egypt; ^2^Molecular Physiology Division, Zoology Department, Faculty of Science, Beni-Suef University, P.O. Box 62521, Beni Suef, Egypt; ^3^Department of Physiology, College of Medicine, Taif University, P.O. Box 11099, Taif 21944, Saudi Arabia; ^4^Department of Pathology, College of Medicine, Taif University, P.O. Box 11099, Taif 21944, Saudi Arabia

## Abstract

Atherosclerosis is a disease in which plaque builds up inside arteries. Cinnamaldehyde (Ci) has many biological properties that include anti-inflammatory and antioxidant activities. Thus, this study was designed to explore the protective effect of Ci against atherosclerosis induced by a high-fat diet (HFD) in Wistar rats. Atherosclerosis was induced by an oral administration of an HFD for 10 weeks. Atherosclerosis-induced rats were supplemented with Ci at a dose of 20 mg/kg bw dissolved in 0.5% dimethyl sulfoxide (DMSO), daily by oral gavage for the same period. Rats were divided into three groups of 10 rats each fed with (a) ND, (b) HFD, and (c) HFD+Ci, daily for 10 weeks. Treatment of rats with Ci significantly reduced the elevated levels of serum total cholesterol (T.Ch), triglycerides (TG), low-density lipoprotein-cholesterol (LDL-Ch), very low-density lipoprotein-cholesterol (VLDL-Ch), and free fatty acids (FFAs) and significantly increased the lowered levels of high-density lipoprotein-cholesterol (HDL-Ch) level. Ci ameliorated the increased cardiovascular risk indices 1 and 2 and the decreased antiatherogenic index. Moreover, Ci reduced the elevated serum creatine kinase (CK), creatine kinase-MB (CK-MB), lactate dehydrogenase (LDH), and aspartate aminotransferase (AST) activities. Ci also improved the heart antioxidant activities by decreasing malondialdehyde (MDA) and increasing glutathione S-transferase (GST), superoxide dismutase (SOD), catalase (CAT), reduced glutathione (GSH), and glutathione peroxidase (Gpx) activities. Furthermore, the supplementation with Ci downregulated the mRNA expression levels of interleukin-1*β* (IL-1*β*), interleukin-6 (IL-6), interleukin-17 (IL-17), and tumor necrosis factor-*α* (TNF-*α*). Thus, Ci successfully elicited a therapeutic impact against atherosclerosis induced by HFD *via* its hypolipidemic, antioxidant, and anti-inflammatory actions.

## 1. Introduction

Diet plays a key role in controlling cholesterol balance, and the consumption of a high-cholesterol diet is regarded as a crucial risk factor for developing hyperlipidemia [1]. Furthermore, a high-fat diet (HFD) leads to excessive lipid accumulation in adipose tissues, which is a crucial factor in the development of hyperlipidemia, obesity, and hepatitis [2]. Hyperlipidemia is a metabolic disorder with a high global prevalence characterized by an elevation in the circulating levels of T.Ch, TG, and LDL-Ch with a reduced HDL-Ch concentration in the blood [3]. High amounts of fat in diets seem to accelerate atherosclerosis development [4]. Atherosclerosis is one of the most widespread conditions that threaten the health and survival of humans. The basic atherosclerosis pathogenesis involves insult damage to the endothelial and smooth muscle cells of arterial walls by various harmful factors including mechanical damage, viral infection, and dyslipidemia, especially abnormally oxidized low-density lipoprotein (oxLDL), resulting in an excessive chronic inflammatory response [5]. Atherosclerosis development is exacerbated by the recurrent and repeated mechanism of oxidative alteration and inflammatory processes translated into a chronic form [6]. Atherosclerosis is a multifactorial disease, whose lipid metabolism dysregulation and aberrant inflammatory responses in the arterial walls at predisposed sites play a central role from initiation to progression and eventually atherosclerotic plaque rupture [7]. The pathological process leads to a progressive accumulation of fibrous elements and lipids in large arteries [8]. A high-fat and high-cholesterol diet results in the increased T.Ch and LDL-Ch levels, and its ability to cause large artery lesions is similar to the discovery of atherosclerosis in humans. A high-cholesterol fat diet containing 2% cholesterol can induce atherosclerotic lesions, and plaques form in the iliac artery and abdominal aorta [9]. Cinnamaldehyde (Ci) occurs naturally in various *Cinnamomum* species; it is used to prepare beverages, medicinal products, perfumes, and cosmetics [10]. It has several biological activities such as hypoglycemic, antihyperlipidemic, antioxidant, and anti-inflammatory [11, 12]. Therefore, this study is aimed at evaluating the protective effect of Ci against HFD-induced atherosclerosis disease in rats.

## 2. Materials and Methods

### 2.1. Experimental Animals

Male Wistar rats (*Rattus norvegicus*), which weighed about 100 ± 10 g and were aged 60 days old, were used as experimental animals. They were obtained from the animal house of Helwan Town, Cairo, Egypt. They were kept under observation for 1 week before the onset of the experiment to be acclimatized and to exclude any intercurrent infection. The chosen animals were housed individually in standard polypropylene cages and maintained under normal atmospheric room temperature (25°C), 55% ± 5% humidity, and 12 h light/12 h dark cycles. Water was provided *ad libitum*. The research protocol and all experimental procedures were approved by the Experimental Animal Ethics Committee of the Faculty of Science for Care and Use of Animals, Beni-Suef University, Egypt (Ethical Approval Number: BSU/FS/2018/2). All precautions were done to decrease the number of animals and minimize the pain, distress, and discomfort of animals.

### 2.2. Dietary Formula and Tested Bioactive Plant Constituents

Diet ingredients, namely, casein, corn starch, cholesterol, and sucrose, were purchased from Oxford Laboratories, Mumbai, India. DL-methionine, bile salts, cellulose, and calcium carbonate were procured from S.D. Fine-Chem Ltd. Mumbai, India; potassium citrate, sodium chloride, and calcium phosphate were bought from Pharmaceutical Chemicals Co. Egypt; corn oil was brought from the supermarket; beef tallow was purchased from the butcher; and vitamin and mineral mix was purchased from the veterinary pharmacy, Beni-Suef, Egypt. The diets were prepared at the Department of Nutrition, Faculty of Veterinary Medicine, Beni-Suef University, Beni Suef, Egypt, at intervals according to the requirements and stored at 4°C until they were used. Ci and its forms were obtained from Carlo Erba Co., Milan, Italy.

### 2.3. Doses and Treatment

The rats were fed with an HFD daily for 10 weeks during the entire study as previously described in our work [13]. Ci was administered by gastric intubation at a dose of 20 mg/kg bw [14] dissolved in 0.5% DMSO daily for 10 weeks during the experimental study.

### 2.4. Experimental Design

**Figure 1:**
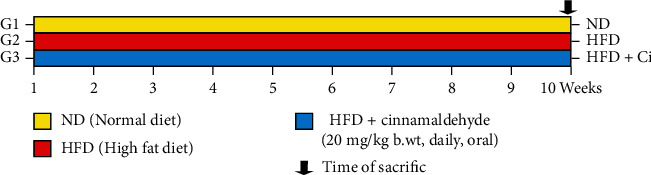
Experimental design for ND (normal diet), HFD (high-fat diet), HFD+Ci (high fat diet+cinnamaldehyde) groups.

After acclimatization for 2 weeks, thirty rats were allocated into three groups, 10 rats in each group: (1) normal diet group: rats fed with a normal diet (ND; 60% starch, 5% corn oil, and 20% casein protein as g %) throughout the experimental period of 10 weeks; (2) HFD group: rats fed with HFD (HFD; 25% sucrose, 40% beef tallow, and 20% casein protein as g %) throughout the experimental period of 10 weeks; (3) HFD+Ci group: rats fed with HFD and administered with Ci throughout the experimental period of 10 weeks. Both group (1) and group (2) were given an equivalent volume from the vehicle (0.5% DMSO) in which Ci was dissolved as shown in Figure 1. At the end of 10 weeks, the rats were sacrificed under mild diethyl ether anesthesia. Blood from each rat was collected from the jugular vein in a centrifuge tube and left to clot for 45 min at room temperature. Sera were separated by centrifugation at 3000 r.p.m. at 30°C for 15 min and kept frozen at −30°C before biochemical investigation. Also, heart tissue samples were excised for histopathological, immunohistochemical, and molecular investigations. Heart tissue samples were rapidly excised and homogenized. Then, 0.5 g of the heart tissue was homogenized in 5 ml of 0.9% sterilized sodium chloride (NaCl; 10% *w*/*v*) by using a Teflon homogenizer (Glas-Col, Terre Haute, USA) to determine oxidative stress and antioxidant defense system.

### 2.5. Biochemical Analysis

Serum T.Ch, TG, and high-density lipoprotein-cholesterol concentrations were determined using reagent kits obtained from the Spinreact Company (Spain). Serum LDL-Ch concentration was determined in accordance with the previously described formula [15]. Serum VLDL-Ch concentration was calculated as follows [16]: VLDL − Ch concentration (mg/dL) = triglycerides/5. Serum-free fatty acid concentrations were estimated as described [17]. Cardiovascular risk indices were determined as follows [18] from the following formulas:

(1) $$\begin{array}{c} \text{Cardiovascular risk index }1=\frac{\text{T}.\text{Ch conc}.}{\text{HDL}‐\text{Ch conc}},\\ \text{Cardiovascular risk index }2=\frac{\text{LDL}‐\text{Ch conc}.}{\text{HDL}‐\text{Ch conc}.}. \end{array}$$

The following equation determined the antiatherogenic index [19]:

(2) $$\begin{array}{c} \text{AAI}=\frac{\text{HDL}‐\text{Ch}\times 100}{\text{T}.\text{Ch}-\text{HDL}‐\text{Ch}}. \end{array}$$

Serum creatine kinase (CK), creatine kinase-MB (CK-MB), and lactate dehydrogenase (LDH) were detected by using kits obtained from HUMAN (Germany). Serum aspartate aminotransferase (AST) was measured using reagent kits purchased from Biosystem S.A., Spain. Malondialdehyde (MDA) was measured as thiobarbituric acid reactive substances (TBARS) according to the method of Ohkawa et al. [20], glutathione S-transferase (GST) activity was assessed according to the method of Habig et al. [21], superoxide dismutase (SOD) activity was assessed according to the method of Nishikimi et al. [22], catalase (CAT) activity was detected according to the method of Aebi [23], reduced glutathione (GSH) content was assayed according to the method of Beutler et al. [24], and glutathione peroxidase (GPx) activity was measured according to the method of Paglia and Valentine [25].

### 2.6. Real-Time Polymerase Chain Reaction (qPCR)

Interleukin-1*β* (IL-1*β*), interleukin-6 (IL-6), interleukin-17 (IL-17), and tumor necrosis factor-*α* (TNF-*α*) gene expressions were determined by real-time polymerase chain reaction (RT-PCR) following the method described previously by Pfaffl [26]. In brief, total RNA was isolated from the heart tissue using the QIAGEN tissue extraction kit (QIAGEN, USA) according to the manufacturer instructions. In brief, the total RNA (0.5–2 *μ*g) was used for cDNA conversion using a high-capacity cDNA reverse transcription kit, Fermentas, USA. Three *μ*l of random primers was added to the 10 *μ*l of RNA which was denatured for 5 minutes at 65°C in the thermal cycler. The primer sequences of the studied genes are as follows: 5′-CACCTTCTTTTCCTTCATCTTTG-3′(forward primer) and 5′-GTCGTTGCTTGTCTCTCCTTGTA-3′(reverse primer) for IL-1*β*, 5′-TGATGGATGCTTCCAAACTG-3′(forward primer) and 5′-GAGCATTGGAAGTTGGGGTA-3′(reverse primer) for IL-6, 5′-GCGCAAAAGTGAGCTCCAGA-3′(forward primer) and 5′-ACAGAGGGATATCTATCAGGG-3′(reverse primer) for IL-17, 5′-ACTGAACTTCGGGGTGATTG-33 (forward primer) and 53-GCTTGGTGGTTTGCTACGAC-3′(reverse primer) for TNF-*α*, and 5′-ATCACCATCTTCCAGGAGCG-3′(forward primer) and 5′-CTGCTTCACCACCTTCTTG-3′(reverse primer) for *β*-actin values were normalized to the quantity of *β*-actin. All the molecular assays were conducted at the Molecular Biology Laboratory, Clinilab, Cairo, Egypt.

### 2.7. Histopathological and Immunohistochemical Investigations

Histopathological investigation was carried out as described previously [27]. In brief, heart samples were fixed for 24 h in 10% neutral formalin buffered, then handled via paraffin-embedding method and dehydrated via ascending grades of ethyl alcohol, clearing in xylene, and immersed in paraffin, then embedded in paraffin wax at 60°C. Sections of 4–5 *μ*m in thickness have been dyed with hematoxylin and eosin. Other heart sections were stained by IL-1*β* and TNF-*α* as described previously [28]. In brief, the heart sections were blocked by incubating in 3% H_2_O_2_ followed by boiling in a citrate buffer, pH 6.0, and blocked using a protein block to avoid nonspecific binding. The sections were then probed with an antibody against IL-1*β* and TNF-*α* washed in phosphate buffer solution and incubated with the secondary antibody. After washing, the sections were counterstained and examined. Histopathological scores were determined for each lesion in ten animals in each group. The lesion score takes four levels. Zero (0) refers to the absence of lesion, “I” indicates a mild degree, “II” indicates a moderate degree, and “III” denotes a severe degree. The free software version of ImageJ (1.51d) was used for immunohistochemical examination and analysis of labeling [29]. Integrated intensities were evaluated using the ImageJ software (in pixels) for positive reactions with IL-1*β* and TNF-*α*.

### 2.8. Statistical Analysis

Data were expressed as mean ± SD. All statistical comparisons were made via one-way analysis of variance (ANOVA) followed by Duncan's method for post hoc analysis using GraphPad Prism software (version 5.01). Mean value is significant at ^∗^*p* < 0.05, ^∗∗^*p* < 0.01, and ^∗∗∗^*p* < 0.001 as compared to the normal diet group and at ^#^*p* < 0.05, ^##^*p* < 0.01, and ^###^*p* < 0.001 as compared to the high-fat diet group. N.S. represents “none significance.” Pearson's correlation coefficient analysis was performed to detect the correlations between different parameters. A total sample size of minimum of 30 rats was estimated for 95% power, with *α*-error probability of 0.05. Sample size was calculated with G∗Power (3.1.9.4) software using a priori analysis with an effect size = 0.76 of contingency tables (one group) using *χ*^2^ tests.

## 3. Results

### 3.1. Effect of Cinnamaldehyde on Serum Lipid Profile, Cardiovascular Risk Indices, and Antiatherogenic Index of Rats Fed with an HFD

**Figure 2:**
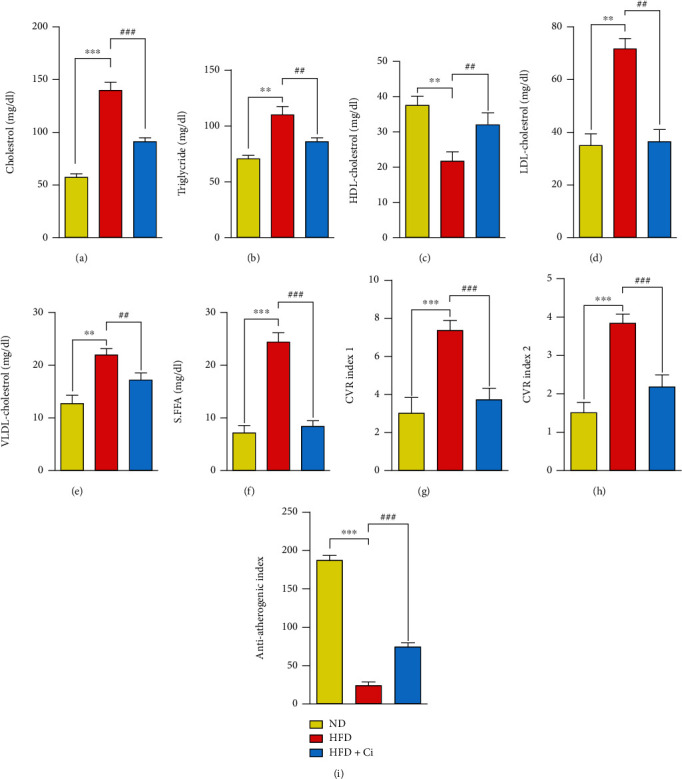
Effect of cinnamaldehyde on the serum lipid profile, CVR indices, and AAI of rats fed with a high-fat diet. The changes in the values of (a) T.Ch, (b) TG, (c) HDL-Ch, (d) LDL-Ch, (e) VLDL-Ch, (f) FFAs, (g) CVR risk 1, (h) CVR risk 2, and (i) AAI among ND (normal diet), HFD (high-fat diet), HFD+Ci (high-fat diet+cinnamaldehyde) groups. Mean value is significant at ^∗^*p* < 0.05, ^∗∗^*p* < 0.01, and ^∗∗∗^*p* < 0.001 as compared to the normal diet group and at ^#^*p* < 0.05, ^##^*p* < 0.01, and ^###^*p* < 0.001 as compared to the high-fat diet group. N.S.: not significant; cholesterol: Ch; LDL-cholesterol: low-density lipoprotein-cholesterol; HDL-cholesterol: high-density lipoprotein-cholesterol; VLDL-cholesterol: very low-density lipoprotein-cholesterol; cardiovascular risk factor 1 (cholesterol/HDL): cholesterol/high-density lipoprotein-cholesterol; cardiovascular risk factor 2 (LDL/HDL): low-density lipoprotein-cholesterol/high-density lipoprotein-cholesterol.

The results revealed that the intake of HFD produced marked impairment, as indicated by a significant increase in serum T.Ch, TG, LDL-Ch, VLDL-Ch, and FFAs and a significant decrease in HDL-Ch compared with normal diet rats. Instead, the oral administration of Ci significantly decreased the elevated levels of serum T.Ch, TG, LDL-Ch, VLDL-Ch, and FFAs and significantly increased the decreased level of HDL-Ch compared to HFD-feeding rats. It also recorded noticeable results compared with those of the normal diet rats as shown in Figures 2(a)–2(f). The effect of Ci on cardiovascular risk indices (CVR1 and CVR2) and the antiatherogenic index (AAI) in HFD-fed rats is illustrated in Figures 2(g)–2(i). The HFD-feeding group demonstrated significant increase in CVR1 and CVR2 compared to normal diet rats. The administration of HFD-fed rats with Ci significantly decreased the high CVR1 and CVR2 and almost reached the normal values. AAI revealed a remarkable decrease compared to the normal-feeding rats. Moreover, the administration of HFD-fed rats with Ci significantly increased the AAI compared with HFD-fed rats.

### 3.2. Effect of Cinnamaldehyde on the Serum Heart Function Enzymes of Rats Fed with a High-Fat Diet

**Figure 3:**
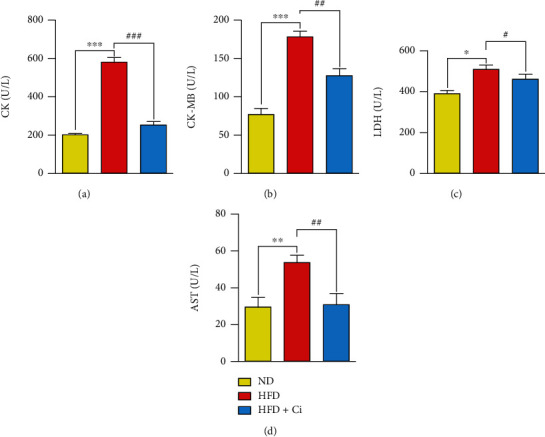
Impact of cinnamaldehyde on serum heart-function enzymes CK, CK-MB, AST, and LDH activities in high-fat-diet-feeding rats. The changes in the values of (a) CK, (b) CK-MB, (c) LDH, and (d) AST among ND (normal diet), HFD (high-fat diet), HFD+Ci (high-fat diet+cinnamaldehyde) groups. Mean value is significant at ^∗^*p* < 0.05, ^∗∗^*p* < 0.01, and ^∗∗∗^*p* < 0.001 as compared to the normal diet group and at ^#^*p* < 0.05, ^##^*p* < 0.01, and ^###^*p* < 0.001 as compared to the high-fat diet group. N.S.: not significant; CK: creatine kinase; CK-MB: creatine kinase-MB; LDH: lactate dehydrogenase; AST: aspartate aminotransferase.

Concerning the impact of Ci on serum heart function enzymes (CK, CK-MB, LDH, and AST), HFD rats exhibited a significant increase in serum CK, CK-MB, AST, and LDH activities compared to the normal diet rat group. Conversely, supplementation of Ci to HFD-feeding rats significantly reduced the elevated serum CK, CK-MB, AST, and LDH activities as illustrated in Figures 3(a)–3(d).

### 3.3. Effect of Cinnamaldehyde on the Heart Oxidative Stress and Antioxidant Defense System of Rats Fed with a High-Fat Diet

**Figure 4:**
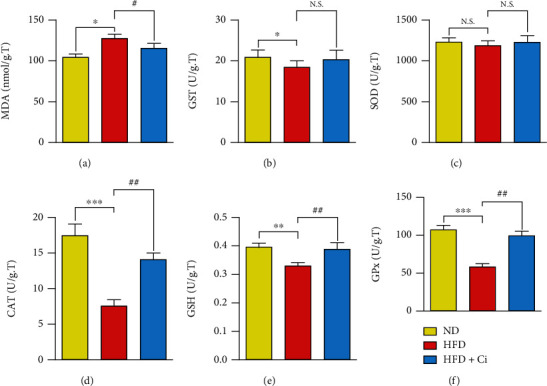
Effect of cinnamaldehyde on the heart oxidative stress and antioxidant defense system of rats fed with a high-fat diet. The changes in the values of (a) MDA, (b) GST, (c) SOD, (d) CAT, (e) GSH, and (f) GPx among ND (normal diet), HFD (high-fat diet), HFD+Ci (high-fat diet+cinnamaldehyde) groups. Mean value is significant at ^∗^*p* < 0.05, ^∗∗^*p* < 0.01, and ^∗∗∗^*p* < 0.001 as compared to the normal diet group and at ^#^*p* < 0.05, ^##^*p* < 0.01, and ^###^*p* < 0.001 as compared to the high-fat diet group. N.S.: not significant; MDA: malondialdehyde; GST: glutathione S-transferase; SOD: superoxide dismutase; CAT: catalase; GSH: reduced glutathione; GPx: glutathione peroxidase.

The results showed that MDA exhibited a significant increase due to HFD administration. In contrast, the treatment of HFD-fed rats with Ci reduced the values back to values comparable to the ones demonstrated in normal diet-fed rats. The rats fed with HFD significantly decreased the GST, SOD, CAT, and GPx activities as well as GSH content compared to those of the normal diet rats. At the same time, Ci treatment exhibited a significant increase in the decreased GST, SOD, CAT, and GP_X_ activities and GSH content compared to those of the corresponding HFD-fed rat group as illustrated in Figures 4(a)–4(f).

### 3.4. Effect of Cinnamaldehyde on IL-1*β*, IL-6, IL-17, and TNF-*α* Inflammatory Biomarkers of Rats Fed with a High-Fat Diet

**Figure 5:**
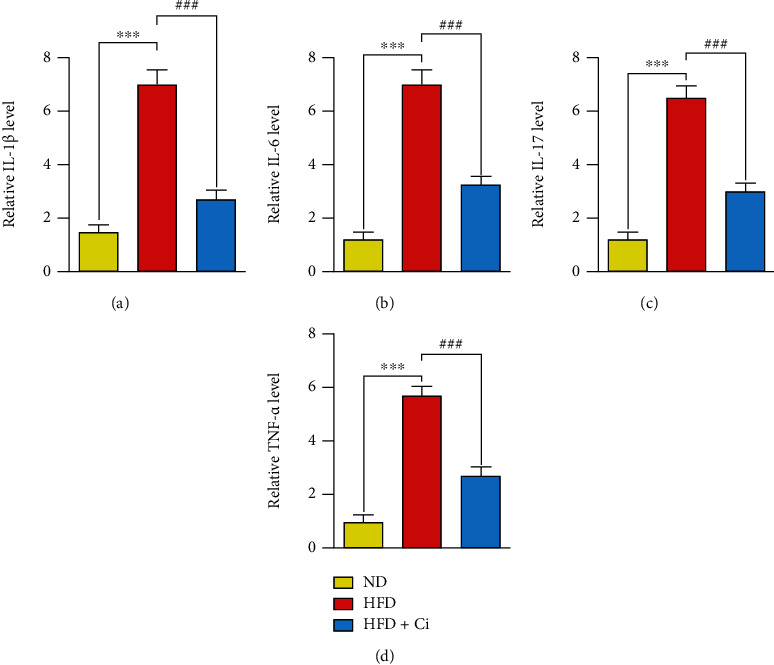
Effect of cinnamaldehyde on IL-1*β*, IL-6, IL-17, and TNF-*α* inflammatory biomarkers of rats fed with a high-fat diet. The changes in the values of (a) IL-1*β*, (b) IL-6, (c) IL-17, and (d) TNF-*α* among ND (normal diet), HFD (high-fat diet), HFD+Ci (high-fat diet+cinnamaldehyde) groups. Mean value is significant at ^∗^*p* < 0.05, ^∗∗^*p* < 0.01, and ^∗∗∗^*p* < 0.001 as compared to the normal diet group and at ^#^*p* < 0.05, ^##^*p* < 0.01, and ^###^*p* < 0.001 as compared to the high-fat diet group. N.S.: not significant; IL-1*β*: interleukin-1*β*; IL-6: interleukin-6; IL-17: interleukin-17; TNF-*α*: tumor necrosis factor-*α*.

Concerning proinflammatory biomarkers, real-time PCR results revealed upregulation in the mRNA gene expression levels of IL-1*β*, IL-6, IL-17, and TNF-*α* in the HFD-feeding rats compared to the normal fat diet group. Conversely, Ci supplementation significantly downregulated the elevated mRNA expression levels of IL-1*β*, IL-6, IL-17, and TNF-*α* compared to that of the HFD-fed rats because of the noticeable amelioration compared to those of the normal ones as shown in Figures 5(a)–5(d).

### 3.5. Histopathological Changes

**Figure 6:**
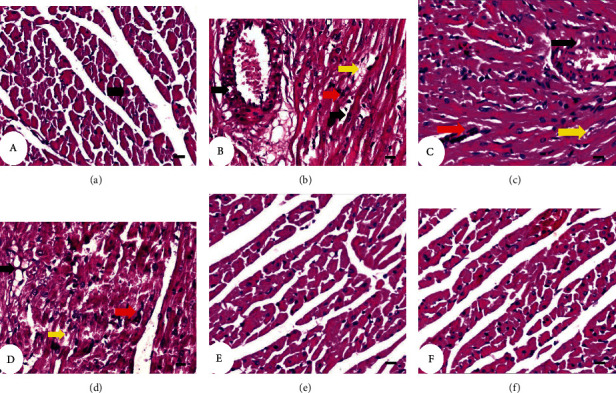
Photomicrograph (a) of cardiac muscle sections of normal diet group showing arranged cardiac muscle fibers with vesicular nuclei (arrow). The fibers are branching and anastomose with each other. Photomicrograph (b) of cardiac muscle sections of HFD-administered group showing degenerated cardiac muscle with deposition of fat (intracytoplasmic fat vacuoles) (yellow arrow), mononuclear cellular infiltration (red arrow), degenerated wall of the congested artery and arterial wall with fat deposition (black arrow), degenerated wall of the congested artery, and focal necrosis and vacuoles in cardiomyocytes (black arrow); (c) cardiac muscle degeneration (yellow arrow) with infiltration of inflammatory cells (red arrow), and myocytes that lost their striations (black arrow) and pyknotic nuclei; and (d) severe degenerated cardiac muscle (yellow arrow) and fragmented cardiomyocytes and some of them lose their nuclei (black arrow), while others reveal pyknotic nuclei and mononuclear cellular infiltration (red arrow). Photomicrographs (e, f) of cardiac muscle sections of HFD-administered rats treated with cinnamaldehyde showed notable heart histological architecture and integrity improvement of the heart. H&E 400x.

The histopathological observations from normal diet rats of the heart tissue showed normal cardiac architecture with striated muscle fibers and centrally located nuclei (Figure 6(a), photomicrograph). HFD-administered rats (Figures 6(b)–6(d); photomicrographs) showed many pathological changes, a degenerated cardiac muscle with deposition of fat (intracytoplasmic fat vacuoles), mononuclear cellular infiltration, arterial wall with fat deposition, degenerated wall of a congested artery, focal necrosis and vacuoles in cardiomyocytes, cardiac muscle degeneration with infiltration of inflammatory cells, myocytes that lost their striations and pyknotic nuclei, and fragmented cardiomyocytes, and some of them lose their nuclei, while others reveal pyknotic nuclei and mononuclear cellular infiltration. Supplementation of HFD-administered rats with cinnamaldehyde (Figures 6(e) and 6(f); photomicrographs) produced marked rescue of these pathological changes. In comparison with HFD-administered control rats, cinnamaldehyde showed notable heart histological architecture and integrity improvement. The histopathological scores for each lesion are depicted in Table 1. The histopathological scores include a degenerated cardiac muscle with deposition of fat (intracytoplasmic fat vacuoles), arterial wall with fat deposition, mononuclear cellular infiltration, degenerated wall of a congested artery, focal necrosis and vacuoles in cardiomyocytes, cardiac muscle degeneration with infiltration of inflammatory cells, and myocytes that lost their striations and pyknotic nuclei. Collectively, all these histopathological changes were remarkably improved to a great extent as a result of the treatment of HFD-administered rats with cinnamaldehyde.

### 3.6. Immunohistochemical Studies

**Figure 7:**
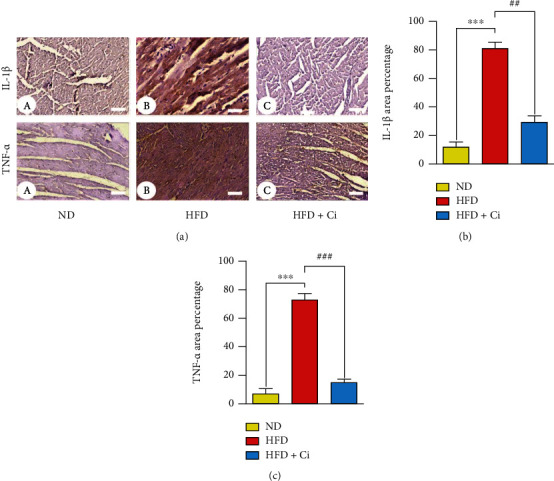
Photomicrograph of interleukin-1*β* (IL-1*β*) and tumor necrosis factor-*α* (TNF-*α*) expression-stained cardiac tissue section of (a) normal diet rats showing negative reaction (IL-1*β* and TNF-*α*; scale bar = 50 *μ*m), (b) HFD-administered rats showing intense brown immunoreaction in the cytoplasm of cardiomyocytes (IL-1*β* and TNF-*α*; scale bar = 50 *μ*m), and (c) HFD-administered rats treated with cinnamaldehyde showing week immunoreactions (IL-1*β* and TNF-*α*; scale bar = 50 *μ*m). Column charts demonstrate the intensity of the brown immunoreaction (b). Mean value is significant at ^∗^*p* < 0.05, ^∗∗^*p* < 0.01, and ^∗∗∗^*p* < 0.001 as compared to the normal diet group and at ^#^*p* < 0.05, ^##^*p* < 0.01, and ^###^*p* < 0.001 as compared to the high-fat diet group. N.S.: not significant.

Immunohistochemical IL-1*β*- and TNF-*α*-stained heart section of normal diet rats showed negative expressions, while the heart of HFD-administered rats showed positive immunoreactions in cardiomyocyte cytoplasm for IL-1*β* and TNF-*α*. On the other hand, HFD-administered rats treated with cinnamaldehyde displayed negative cytoplasmic immunoreactions for IL-1*β* and TNF-*α* (Figure 7(a)).

### 3.7. Correlations between CK-MB, AAI, and Cholesterol with IL-1*β*, IL-6, IL-17, TNF-*α*, MDA, and SOD of Rats Fed with a High-Fat Diet

Regarding the correlation between HFD and HFD administered with Ci CK-MB showed positive correlations with each of IL-1*β* (*r*, 0.800; *p* > 0.01), IL-6 (*r*, 0.807; *p* > 0.001), IL-17 (*r*, 0.845; *p* > 0.05), TNF-*α* (*r*, 0.748; *p* > 0.001), and MDA (*r*, 0.612; *p* > 0.001). However, a negative correlation was observed with SOD (*r*, -0.719; *p* > 0.05) (Supplementary Figures 1A-F). AAI showed positive correlation with SOD (*r*, 0.532; *p* > 0.01). Nonetheless, a negative correlation was observed with IL-1*β* (*r*, -0.615; *p* > 0.05), IL-6 (*r*, -0.817; *p* > 0.05), IL-17 (*r*, -0.624; *p* > 0.05), TNF-*α* (*r*, -0.615; *p* > 0.001), and MDA (*r*, -0.556; *p* > 0.0) (Supplementary Figures 1G-L). Cholesterol indicated positive correlations with IL-1*β* (*r*, 0.801; *p* > 0.05), IL-6 (*r*, 0.735; *p* > 0.01), IL-17 (*r*, 0.889; *p* > 0.05), TNF-*α* (*r*, 0.669; *p* > 0.05), and MDA (*r* 0.889; *p* > 0.05). Conversely, a negative correlation was observed with SOD (*r*, -0.704; *p* > 0.01) (Supplementary Figures 1M-R). Altogether, the data may suggest a strong association between hyperlipidemia, oxidative stress, and inflammation in atherosclerosis disease.

## 4. Discussion

**Figure 8:**
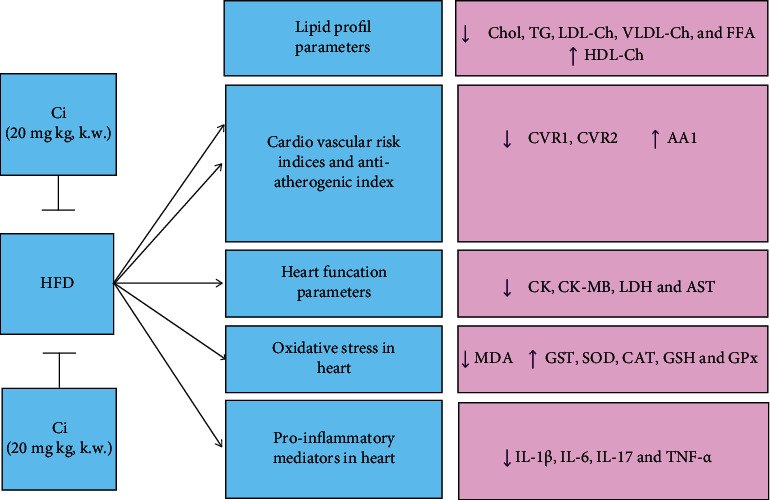
Model of work showing the mitigation effect of cinnamaldehyde against high-fat-diet-induced atherosclerosis.

Our results could be discussed in two main ways: first, the development of an atherosclerosis disease model induced by feeding with an HFD, and second, the ameliorative effects of Ci against changes induced by HFD as illustrated in Figure 8. The body weight excess was associated with the increased plasma levels of LDL-Ch, TG, and fasting glucose and decreased HDL-Ch [30]. It has been revealed that the serum T.Ch, TG, VLDL-Ch, LDL-Ch, and phospholipid concentrations significantly increased. However, the HDL-Ch level significantly decreased in the rat groups with obesity [31]. Serum lipid abnormalities are an important characteristic of diabetic rats fed with HFD, and they include higher serum cholesterol and TG levels, resulting from the increased mobilization of FFAs to central blood circulation from peripheral deposits. High T.Ch and TG levels in the blood may be due to the increased absorption of dietary cholesterol from the small intestine and increased formation and absorption of TG in chylomicrons that form after the consumption of exogenous fat-rich diet or decreased TG uptake in peripheral tissues and through increased endogenous TG-enriched hepatic VLDL production under a diabetic condition [32]. In different obese states, serum cholesterol level frequently increases, possibly because of the decreased level of HDL-Ch, together with an increased LDL-Ch concentration [31]. Hypertriglyceridemia caused by HFD has been reported to be due to the increased hepatic TG production and VLDL-Ch secretion due to the increased adipocyte hormone-sensitive lipase activity and the decreased lipoprotein lipase activity in muscles [33]. High blood TG concentrations in the form of VLDL tend to accompany this condition and activate cholesterol ester transfer protein, leading to an increased transfer of TG from VLDL to HDL, a subsequent increase in HDL clearance, and the decreased HDL concentration [34]. Serum FFAs are elevated by accelerated lipolysis in the peripheral adipose tissue and visceral fat in the state of IR. Excessive inappropriate dietary fat intake combined with peripheral IR continues TG hydrolysis through lipoprotein lipase and other genetic alterations in key lipid metabolic pathways, leading to increased blood FFA concentration and resulting in excessive muscle fat accumulation and increased liver TG and cholesterol ester concentration [35]. This excess in FFAs drives the overproduction of TG-rich lipoprotein particles including LDL-Ch and VLDL-Ch; a reciprocal decrease in HDL accompanies the hypertriglyceridemia characteristic of type 2 diabetes [36]. The fatty acid profile diet plays a crucial role in IR [37]. The loss of insulin action causes a shift in balance from FFA oxidation to esterification, resulting in the elevated secretion of VLDL [31]. FFA mobilization occurs faster in visceral fat and subsequently increases the FFA levels in systemic circulation compared to subcutaneous fat. This FFA excess may cause the enhancement of lipid synthesis and gluconeogenesis and IR leading to hyperlipidemia, hypertension, glucose intolerance, and atherosclerosis [38]. Our results showed that the administration of Ci significantly ameliorates serum lipid profile parameters. These findings are consistent with [39] who stated that Ci treatment significantly decreases T.Ch, TG, VLDL-Ch, and LDL-Ch levels and increases HDL-Ch levels. The Ci-treated group significantly improved its lipid profile because it enhances lipolysis in adipose tissues [40]. An increase in the HDL-Ch level after Ci administration may be due to the activity of lecithin cholesterol acyltransferase, contributing to blood lipid regulation [41]. Predictive indices such as the atherogenic index of plasma (AIP) have been developed to estimate the CVD risk [42]. It is associated with VLDL, HDL, and LDL particle sizes and predicted CVD risk [43]. AIP is the most sensitive marker among other atherogenic indices such as the atherogenic coefficient (TC-HDL-Ch/HDL-Ch), Castelli's risk index I (T.Ch/HDL-Ch), and Castelli's risk index II (LDL-Ch/HDL-Ch) [44]. In the present study, the increased levels of cardiovascular risk index 1 (CVR1) and cardiovascular risk index 2 (CVR2) and the decreased level of the AAI in HFD agree with [39] which revealed that obesity has a direct relationship with an increase in the blood atherogenic index that ultimately predicts CVD. A previous study hypothesized that higher levels of T.Ch and TG are crucial factors in lipoprotein metabolism. Its higher concentration is attributed to the increased formation and LDL deposition, which is potently atherogenic [45]. Treatment with Ci significantly ameliorated the elevated CVR1 and CVR2 and the decreased AAI. These results were consistent with previous findings [46], suggesting that Ci inhibited the FMO3 enzyme, thereby reducing the atherosclerosis risk.

CK is an enzyme, catalyzing the adenosine triphosphate- (ATP-) dependent phosphorylation of creatine, important for tissue energy buffering with variable energy demands, most notably in cardiac and skeletal muscles. Serum CK and LDH levels were used as biomarkers for diagnosing MI and were widely utilized as markers of tissue damage and may give information on the cardiac tissue state [47]. CK-MB has greatly revolutionized the diagnosis and management of acute myocardial infarction (MI) [48]. When cells are damaged by oxidative stress or destroyed because of deficient oxygen supply or glucose, the cell membrane becomes permeable or ruptures, leading to enzyme leakage. This enzyme then enters the bloodstream, increasing its serum concentration [49]. In the present study, serum CK, CK-MB, LDH, and AST activities significantly increased due to the administration of HFD, reflecting impairment in heart function. These results agree with [50] which revealed that obese rats significantly increased serum CK, CK-MB, and LDH activities compared with those of normal rats. The reported increase in the relative heart weight is associated with a remarkable increase in CK activities, reflecting myocardial injury [51]. When myocardial cells are injured, many enzymes such as CK-MB, LDH, AST, and ALT can be released from myocardial cells to the extracellular fluid because of alterations in plasma membrane integrity and permeability [52]. Dietary fat is one of the most important environmental factors associated with CVD incidence; diets with high cholesterol and saturated fat promote atherosclerosis [53]. Serum CK, CK-MB, LDH, and AST activities remarkably decreased in rats fed with an HFD and treated with Ci compared with rats with an HFD. These results agreed with [54], who showed that Ci has an anti-inflammatory and antioxidative activity to relieve heart injury in metabolic syndrome, and Ci alleviates the ischemic myocardial injury of rats. Therefore, Ci-elevated serum HDL-Ch potentially prevented the development of atherosclerosis and coronary heart disease, which are common secondary complications of gestational diabetes mellitus (GDM) [55].

Oxidative stress and inflammatory progress are correlated and mediate the development of atherosclerotic changes in a rat model with hyperlipidemia [56]. Growing evidence supports that increased oxidative stress is attributed to excessive free radicals that interplay between atherosclerosis and hypercholesterolemia [57]. The oxidative modification of LDL plays an immense role in the initial atherosclerosis development and promotes the further accumulation of free radicals in arterial walls [58]. Oxidative stress results from excess circulatory lipids that have accelerated in the early atherosclerosis stage [59]. In the present study, feeding with HFD caused a significant elevation in cardiac LPO level. Moreover, our results disclosed that CK-MB exhibited a positive correlation with MDA, while a negative correlation was detected with CK-MB and SOD indicating that oxidative stress associated with hyperlipidemia may contribute to the development of atherosclerosis. Furthermore, oxLDL and MDA levels were high, suggesting high oxidative stress and concomitant high T.Ch and LDL levels; these findings might explain the atherosclerotic plaque present in the HFD group [58]. The aortic MDA and oxLDL levels in the HFD group significantly increased, indicating an excessive oxidative stress formation in the aortic tissue following the HFD ingestion as a consequence of lipid oxidation [60]. In a condition with excess dietary fat ingestion, lipid constituents, particularly LDL-Ch, are highly permeable to the subendothelial layer [61]. Oxidative modification occurred and transformed the LDL into an oxidative form. This finding consequently increases MDA formation, prompting more macrophages to engulf excess lipids as a part of a clearing process [62]. Similarly, overweight- or obesity-induced cardiac dysfunction is associated with excessive oxidative stress, mitochondrial ROS, and massive cardiac cell loss [63]. The potential mechanism of elevated cardiac tissue lipid peroxidation may be caused by an increased lipid substrate within the myocardium that serves as a larger oxidation target by free radicals [64]. Lipid peroxidation increases and inactivates enzymes by cross-linking with MDA; consequently, the accumulation of hydrogen peroxide (H_2_O_2_), superoxide, and hydroxyl radicals increases, further stimulating lipid peroxidation. This finding confirmed and supported the concept of antioxidant enzyme and protein inactivation by a high lipid peroxidation level in obesity [65]. Circulating concentrations of FFAs and lipids increase after HFD feeding, and hyperlipidemia and elevated plasma LDL may initiate atherosclerosis [66]. Our results agreed with a previous finding [67], which suggested that HFDs trigger several metabolic perturbations, including dyslipidemia, by altering the proportion of VLDL/LDL and HDL; as a result, oxidative stress and mitochondrial dysfunction occur.

In our study, Ci administration to rats fed with an HFD resulted in decreased levels of cardiac MDA and increased GST, SOD, CAT, GSH, and GPx activities. These results are consistent with [54]. The CAT and GPx activities of the HFD group were significantly lower than those of the normal group likely because of the suppression or deactivation of these enzymes by oxidative stress [58]. Similarly, *C. cassia* (Chinese cinnamon) is remarkable for its function as a spice and its antioxidant activity. It also has potential therapeutic benefits in CVD, and pretreatment with cinnamic acid or cinnamaldehyde increases the activity of SOD and decreases the levels of MDA in the myocardium [68]. Inflammatory cytokines actively participate in atherosclerosis pathogenesis. The present study showed a positive correlation between hyperlipidemia, oxidative stress, and inflammation confirming the relationship between hyperlipidemia, inflammation, and oxidative stress in the HFD-induced atherosclerosis rats. We may conclude that this significant correlation may be associated with heart injury and contribute to the development of cardiomyopathy. Saturated fatty acids in lard, the main fat source in HFD, may contribute to the induction of proinflammatory markers [69]. Feeding with HFD increased the circulating proinflammatory cytokine [70]. An increase in circulating proinflammatory cytokines and a decrease in anti-inflammatory cytokines/adipokines are key events of obesity induced by diet and associated with metabolic endotoxemia; these conditions further promote IR and low-grade systemic inflammation [71]. In the present study, Ci administration to rats fed with an HFD resulted in a significant decrease in IL-1*β*, IL-6, IL-17, and TNF-*α* mRNA expression levels. Consistent with our results, the previous finding demonstrated that cinnamic acid and cinnamaldehyde decrease the serum IL-6 and TNF-*α* levels, suggesting that their cardioprotective effects were associated with anti-inflammatory properties [72]. Ci exerts anti-inflammatory effects in short-term pretreatments by blocking the inhibitory protein I*κ*B-*α* degradation; conversely, it does so by inducing NF-E2-related factor 2- (Nrf2-) associated genes, including heme oxygenase-1, during long-term pretreatments [73]. It reduces ROS production and the secretion of IL-1*β* to alleviate inflammation associated with metabolic disturbance in murine RAW 264.7 or J774A.1 macrophages; it suppresses plasma TLR4 expression and myocardium inflammatory cell infiltration from mice with viral myocarditis. A study has shown that the antioxidative effect of Ci and the redox balance restoration are responsible for its anti-inflammatory effect [74]. The anti-inflammatory effects of Ci and eugenol are observed because of their ability to inhibit proinflammatory cytokine secretion and enhance anti-inflammatory IL-10 secretion, thereby regulating Th1 and Th2 balance [75]. In the present study, the histopathological changes due to HFD administration showed many pathological changes including a degenerated cardiac muscle with deposition of fat (intracytoplasmic fat vacuoles), mononuclear cellular infiltration, arterial wall with fat deposition, degenerated wall of the congested artery, focal necrosis and vacuoles in cardiomyocytes, cardiac muscle degeneration with infiltration of inflammatory cells, myocytes that lost their striations and pyknotic nuclei, and fragmented cardiomyocytes, and some of them lose their nuclei, while others reveal pyknotic nuclei and mononuclear cellular infiltration. Our results are consistent with [76], whose study showed that mast cell activation and inflammatory cell infiltration in the cardiac tissue of HFD-induced obese rats and exercise decreased mast cell activation and inflammatory cell infiltration. Activated mast cells release many chemokines, histamine, proteases, and proinflammatory cytokines. Mast cell-derived TNF-*α* promotes the upregulation of IL-6 in infiltrating leukocytes and triggers the cytokine cascade responsible for the induction of intercellular adhesion molecule 1 by myocytes, following neutrophil-induced injury [77]. Abnormal lipid metabolism is associated with impaired mitochondrial structure and function, cardiac efficiency loss, and cardiomyopathy, particularly due to lipid-induced apoptosis. Previous studies stated that the produced ROS could lead to mitochondrial dysfunction and cardiac hypertrophy [78]. Inflammatory cytokines are highly expressed throughout atherosclerosis, accelerating the initiation and progression of atherosclerotic lesions [79]. TNF-*α* was considered a proinflammatory cytokine, resulting in an inflammatory disorder during multiple steps of atherosclerosis [80]. IL-6 has also been discovered as another risk factor in chronic inflammatory and atherosclerotic plaque development [81]. Meanwhile, the levels of inflammatory cytokines in serum are used as important indicators of atherosclerosis [11]. Ci potentially amended the histopathological deterioration produced by HFD since cardiac muscle showed nearly normal architecture. This amelioration in heart histopathological architecture is agreeable with [68]. Also, another study revealed that Ci exerted antiatherosclerotic effects and reduced inflammation in HFD-induced ApoE−/− mice and inhibited MDA production. Moreover, this study indicated that the improvement of atherosclerosis by Ci might be closely associated with the decrease in the serum lipid and lipid peroxidation levels, and it also alleviated the inflammation in atherosclerosis by reducing inflammatory factors [11]. In conclusion, cinnamaldehyde has several biological activities such as hypoglycemic, antihyperlipidemic, antioxidant, and anti-inflammatory. It may elicit a beneficial therapeutic effect against atherosclerosis induced by a high-fat diet *via* modulation of hyperlipidemia, oxidative stress, and inflammation.

## Figures and Tables

**Table 1:** Histological lesion scores of the heart in the normal diet, HFD-administered group, and HFD-administered group supplemented with cinnamaldehyde.

Histological changes	Score	Normal diet	High-fat diet	High-fat diet+cinnamaldehyde
Degenerated cardiac muscle with deposition of fat (intracytoplasmic fat vacuoles)	0	10 (100%)	1 (10%)	100 (100%)
	I	—	1 (10%)	—
	II	—	5 (50%)	—
	III	—	3 (40%)	—
Arterial wall with fat deposition	0	10 (100%)	1 (10%)	8 (80%)
	I	—	4 (40%)	2 (20%)
	II	—	3 (30%)	—
	III	—	2 (20%)	—
Mononuclear cellular infiltration	0	10 (100%)	1 (10%)	9 (90%)
	I	—	3 (30%)	1 (10%)
	II	—	3 (30%)	—
	III	—	3 (30%)	—
Degenerated wall of congested artery, focal necrosis, and vacuoles in cardiomyocytes	0	10(100%)	—	10 (100%)
	I	—	3 (30%)	—
	II	—	3 (30%)	—
	III	—	4 (40%)	—
Cardiac muscle degeneration with infiltration of inflammatory cells and myocytes that lost their striations and pyknotic nuclei	0	9 (90%)	—	10 (100%)
	I	1 (10%)	2 (20%)	
	II	—	4 (40%)	—
	III	—	4 (40%)	—

0: absence of lesion; I: mild; II: moderate; III: severe. The number of animals in each group is 10. The % in parentheses is the percent of animals in each grade.

## Data Availability

Data are available and accessible under reasonable request.

## Supplementary Materials

Supplementary Materials Figure 1: correlations between CK-MB, antiatherogenic index, and cholesterol with IL-1*β*, IL-6, IL-17, TNF-*α*, MDA, and SOD among high-fat-diet-feeding rats.
