# Supplementary material for: Cinnamaldehyde Mitigates Atherosclerosis Induced by High-Fat Diet via Modulation of Hyperlipidemia, Oxidative Stress, and Inflammation
Source: Oxid Med Cell Longev. 2022 Jun 21;2022:4464180. doi: 10.1155/2022/4464180 (PMC9239836; doi:10.1155/2022/4464180)
Supplement: Supplementary Materials — Figure 1: correlations between CK-MB, antiatherogenic index, and cholesterol with IL-1β, IL-6, IL-17, TNF-α, MDA, and SOD among high-fat-diet-feeding rats. [file 4464180.f1.pdf]

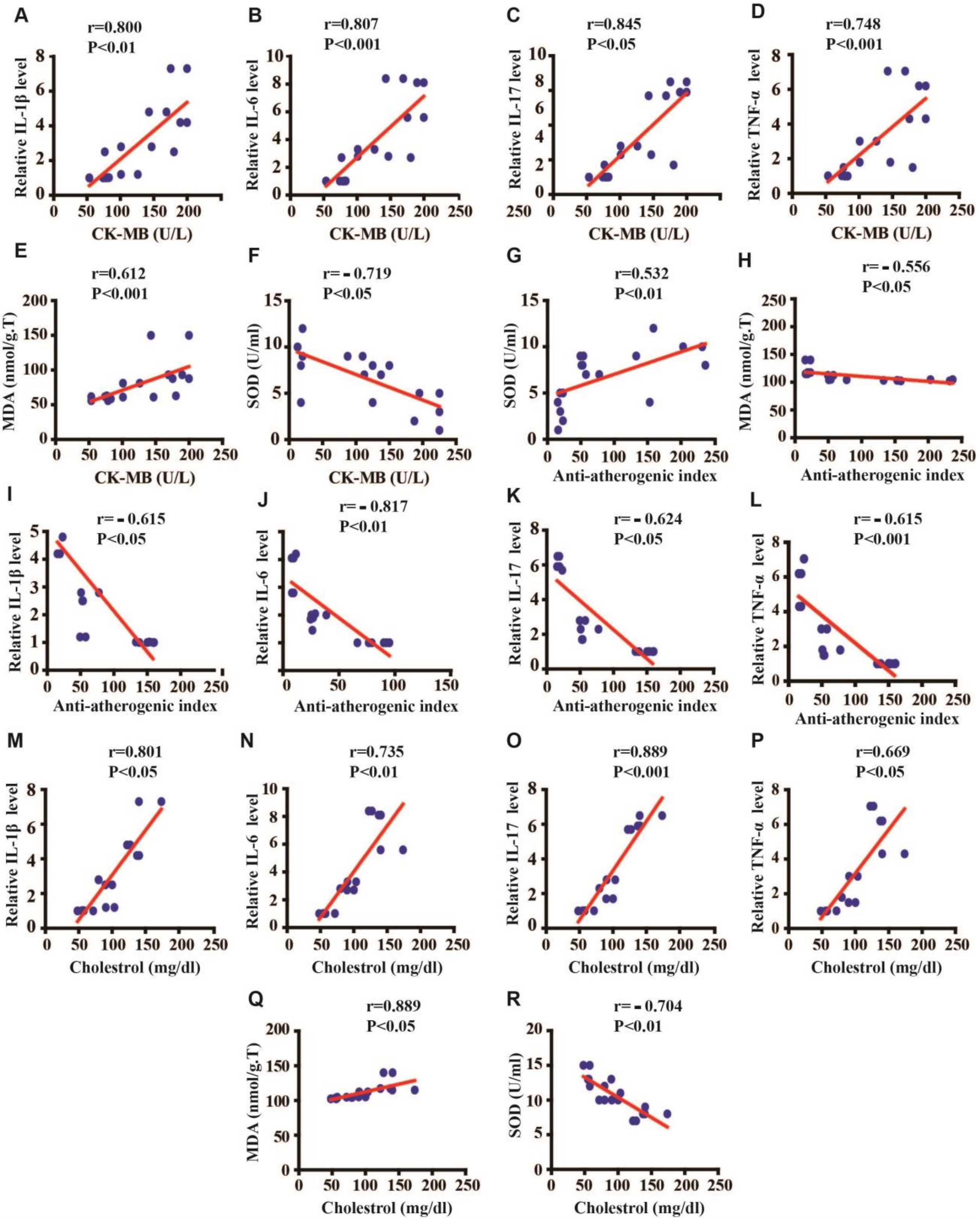

Fig. 1: Correlations between CK-MB, anti-atherogenic index and cholesterol with IL-1β, IL-6, IL-17, TNF-α, MDA and SOD among high-fat diet feeding rats
